# Combining Video Magnification with Machine Learning-Based Source Identification for Contactless Heart Rate Monitoring

**DOI:** 10.3390/s26092706

**Published:** 2026-04-27

**Authors:** Tiago de Avelar, Vicente M. Garção, Hugo Plácido da Silva

**Affiliations:** 1 Department of Bioengineering, Instituto Superior Técnico, Av. Rovisco Pais 1, 1049-001 Lisboa, Portugal; vicente.garcao@tecnico.ulisboa.pt (V.M.G.); hsilva@lx.it.pt (H.P.d.S.); 2 Instituto de Telecomunicações, Av. Rovisco Pais 1, 1049-001 Lisboa, Portugal; 3 Lampsy Health, Av. das Forças Armadas 107, 6E, 1600-078 Lisboa, Portugal

**Keywords:** contactless monitoring, heart rate, computer vision, machine learning, blind source separation

## Abstract

Conventional contact-based monitoring of heart rate (HR) presents challenges such as patient discomfort, skin irritation, and poor long-term adherence, motivating the development of contactless, video-based sensing systems. This study proposes a robust hybrid framework combining advanced signal processing with machine learning to enhance HR estimation accuracy from facial video. The methodology integrates a two-stage geometric stabilization pipeline with dense facial tessellation to mitigate motion. Eulerian Video Magnification (EVM) amplifies subtle color variations, followed by chrominance-based Region of Interest (ROI) filtering. Signal recovery utilizes a sliding-window Principal Component Analysis (PCA) for local coherence, followed by Second-Order Blind Identification (SOBI), with a Light Gradient Boosting Machine (LightGBM) classifier employed to automatically identify physiological sources. Validated on the challenging COHFACE dataset, the approach achieves a Mean Absolute Error (MAE) of 1.50 bpm, a Root Mean Square Error (RMSE) of 3.07 bpm, and a Pearson Correlation Coefficient (PCC) of 0.97 on the test set. The method demonstrates robustness across diverse lighting conditions, outperforming traditional algorithms and achieving parity with state-of-the-art deep learning models, while offering an interpretable solution for contactless health monitoring.

## 1. Introduction

Cardiovascular monitoring plays a critical role in the early detection and management of conditions that impose substantial burdens on individuals and healthcare infrastructure [1,2]. Among the fundamental vital signs, heart rate (HR), measured in beats per minute (bpm), serves as a key indicator of cardiac function and overall physiological state [3].

The adoption of wearable technologies for cardiac monitoring has expanded considerably, offering continuous tracking capabilities in both clinical and home environments [2]. These devices provide clinicians and patients with granular physiological data that facilitate informed decision-making and personalized care strategies [4,5]. Nevertheless, commercial wearables depend on sustained skin contact through chest straps, adhesive electrodes, or wrist-worn sensors, which may lead to dermatological reactions, restricted mobility, and user fatigue over extended monitoring periods. Such issues are particularly pronounced in pediatric and geriatric populations [1,5,6]. Combined with concerns regarding data privacy, device maintenance, and lifestyle disruption, these limitations frequently result in diminished compliance and eventual device abandonment [5,7,8].

These challenges have motivated research into contactless sensing paradigms, often conceptualized as invisible monitoring systems that operate unobtrusively within users’ everyday environments [9,10]. By eliminating direct contact with the skin, such approaches circumvent issues related to discomfort, stigmatization, and infection transmission. This is of particular importance, e.g., for immunocompromised patients, neonates, and individuals requiring long-term surveillance. Furthermore, contactless systems enable deployment across diverse settings, from intensive care units to residential spaces, thereby enhancing accessibility and continuity of care [1,5,6].

This study presents a contactless methodology for accurate HR estimation from conventional video recordings, addressing the limitations of contact-based and wearable monitoring systems. The proposed approach aims to implement a hybrid approach that combines advanced signal processing techniques with machine learning to robustly extract cardiac information from facial video. To the best of our knowledge, while Eulerian Video Magnification (EVM) and Independent Component Analysis (ICA) have been employed across multiple independent works, no study has demonstrated the effectiveness of integrating both within this specific sequential manner. Therefore, the primary novel contribution of this work is the complete, end-to-end design of this structured, interpretable pipeline for contactless heart rate monitoring.

The primary contributions of this work are as follows:

(i) A novel contactless method for heart rate estimation utilizing Eulerian Video Magnification (EVM) combined with multi-patch facial analysis.
(ii) A multi-patch framework exploring the physiological information from distinct facial regions, rather than treating the face as a single homogeneous Region of Interest (ROI).
(iii) A comprehensive signal processing pipeline integrating Principal Component Analysis (PCA) and Second-Order Blind Identification (SOBI) for dimensionality reduction and artifact suppression, complemented by automated physiological source identification through a machine learning classifier trained on frequency-domain and morphological signal characteristics.
(iv) Empirical validation using the publicly available COHFACE dataset, demonstrating performance comparable to the current deep learning state of the art.

## 2. Background and Related Work

**Figure 1:**
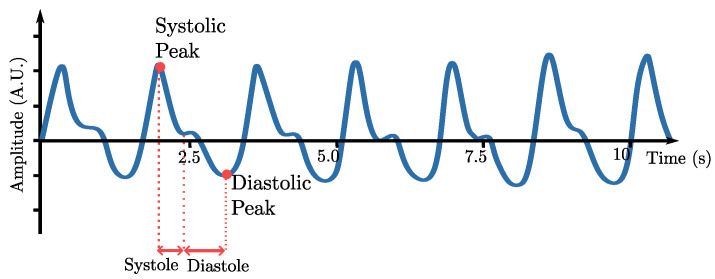
Schematic representation of a PPG waveform illustrating systolic and diastolic peaks. The interval between successive systolic peaks (peak-to-peak time) corresponds to the cardiac period, from which HR can be derived. Note that considerable inter-subject variability exists in the waveform shape.

Photoplethysmography (PPG) emerges as a widespread non-invasive optical technique used to assess or monitor pulsatile changes in blood volume within peripheral vascularized tissues during each cardiac cycle, making it a primary method for HR measurement [3]. This physical principle relies on the variation in light absorption capacity exhibited by tissues throughout the cardiac cycle. A cardiac cycle encompasses both systolic and diastolic phases, with diastole corresponding to ventricular filling, while systole involves the pumping of blood, including its propagation to peripheral tissues [3]. This spike in blood volume leads to increased light absorption by the tissues compared to the diastolic phase [3]. This variation uncovers the pulsatile waveform observed in PPG signals [3,11]. A representative PPG waveform is shown in Figure 1.

Advances in imaging technologies have extended this principle to contactless measurement through remote Photoplethysmography (rPPG), in which standard video cameras capture subtle photoplethysmographic signals remotely [12]. The underlying physiological mechanism remains consistent between contact and contactless approaches, namely hemodynamic changes associated with cardiac pulsation induce measurable variations in optical signals, from which HR and other cardiovascular parameters can be extracted [12].

RGB imaging systems have become the predominant modality for facial video acquisition in rPPG applications, offering greater flexibility in deployment compared to specialized sensing modalities such as near-infrared imaging, radar, or ultrasound [13].

**Figure 2:**
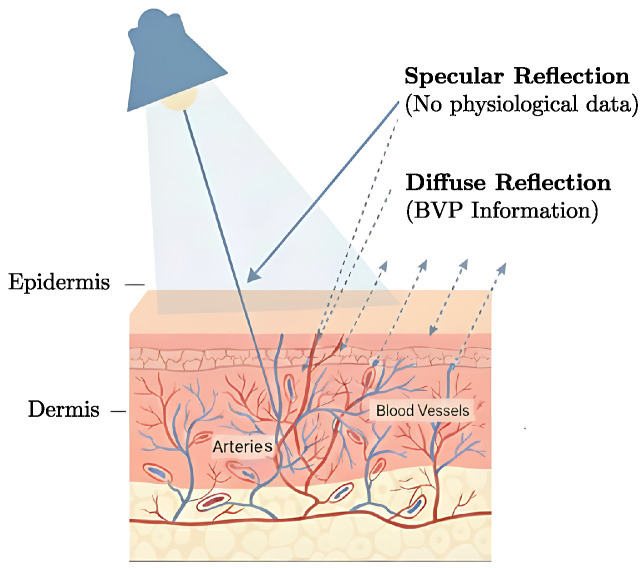
Illustration of the dichromatic reflection model of the skin. Incident light on the skin surface produces two components: specular reflection, which contains no physiological information, and diffuse reflection, which penetrates the epidermis and dermis, interacting with subcutaneous blood vessels and carrying photoplethysmographic information used in rPPG. Adapted from [14].

The interaction between incident light and skin tissue follows the dichromatic reflection model, which distinguishes two reflection mechanisms with fundamentally different information content [14] (Figure 2).

Specular reflection occurs at the air–skin interface, producing mirror-like reflections devoid of physiological information, whereas diffuse reflection results from photons that penetrate the epidermal and dermal layers before backscattering, thereby carrying cardiovascular pulsations [14,15].

This distinction necessitates careful consideration in the Region of Interest (ROI) selection, as areas dominated by specular components yield degraded signal quality [14]. The heterogeneous anatomical structure of facial tissue adds complexity; variations in skin thickness and vascular density across regions result in spatially non-uniform signal characteristics [14]. Commonly investigated ROIs include the forehead and cheek regions, each presenting distinct signal-to-noise characteristics [14]. Despite this spatial heterogeneity, most existing approaches treat the face holistically, overlooking the potential benefits of sub-regional analysis for improved signal extraction [16].

A structured summary of representative approaches for video-based HR estimation, including their objectives, methodologies, performance, and limitations, is provided in Table 1.

## 3. Methods

### 3.1. Dataset

The HR estimation experiments were conducted using the publicly available COHFACE dataset [24]. The dataset comprises 160 videos of 40 subjects (12 females and 28 males) recorded at a resolution of 640 × 480 pixels and 20 FPS, under both natural and studio lighting conditions.

Each video is accompanied by a reference PPG signal, sampled at 256 Hz, which serves as the ground truth for HR estimation. Signals were first detrended by removing their mean and applying the method proposed by Tarvainen et al. [25], using a regularization parameter λ= 120. The resulting signals were bandpass-filtered with a third-order Butterworth filter within the cardiac frequency range (0.8–3 Hz) and resampled to 20 Hz. Hilbert-based normalization was applied, to standardize the amplitude envelope of the PPG signal. The signal was segmented into 15 s segments and the peak frequency of the FFT multiplied by 60 was taken as the reference HR for that segment.

### 3.2. Proposed Approach Overview

The proposed methodology for contactless HR estimation comprises a sequence of processing stages designed to robustly extract the rPPG signal from facial video data. The process begins with video preprocessing, including dense facial landmark detection and a two-stage geometric stabilization to mitigate motion artifacts. Spatially redundant ROIs are subsequently filtered based on illumination quality, after which EVM is applied to amplify subtle cardiac-related color variations. The resulting rPPG time series are then cleaned through outlier removal and harmonic suppression. Subsequently, a multi-stage approach based on sliding-window PCA and SOBI is employed to isolate the physiological source of interest. A machine learning classifier is then used to automatically select the independent component corresponding to the cardiac signal. Finally, the HR is estimated from the reconstructed final signal. The following sections provide a detailed description of each stage.

**Figure 3:**
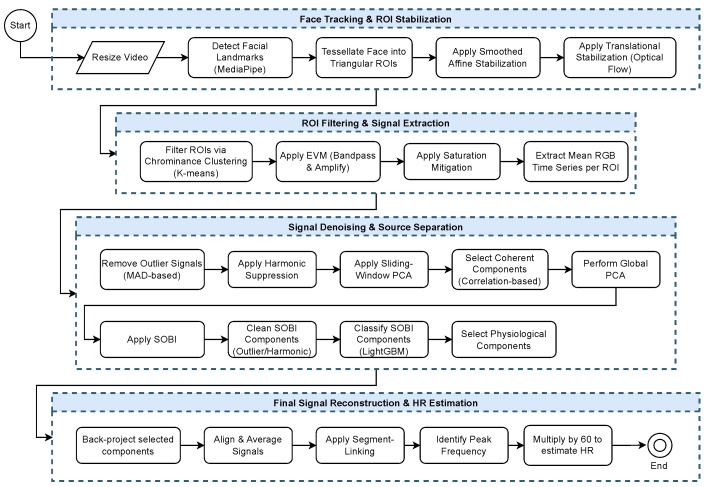
Flowchart of the proposed approach for contactless HR measurement from facial video. The process consists of four main stages: face tracking and ROI stabilization; ROI filtering and signal extraction; signal denoising and source separation, followed by final signal reconstruction and HR estimation.

A flowchart of the methodology developed in this work is illustrated in Figure 3.

### 3.3. Preprocessing and Dense Facial ROI Extraction

Input videos were resized to a fixed resolution of 320 × 240 pixels. Facial landmarks were detected with the MediaPipe Face Mesh model, which provided a dense set of 478 facial landmarks.

**Figure 4:**
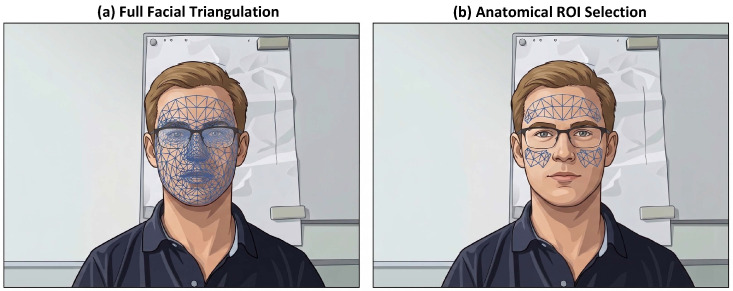
ROI selection process. Candidate ROIs for signal extraction were defined through a two-step procedure. (**a**) A dense triangular mesh was generated from the 478 facial landmarks provided by the MediaPipe framework. (**b**) The mesh was then filtered to retain only triangles whose centroids fell within predefined anatomical regions (the forehead and the cheeks).

Rather than collapsing the facial region into a single averaged patch, as commonly done in earlier work, the face (to protect subject privacy, figures depicting faces have been replaced with illustrations, as explicit publication consent was not provided by the dataset source) was tessellated into multiple small triangular regions based on a predefined triangulation over the 478 landmarks (Figure 4a). A triangle was included among the candidate ROIs if its centroid (computed in the canonical reference space) fell within the anatomical polygons corresponding to the forehead or cheeks, resulting in a set of triangles per subject for the entire video (Figure 4b).

Two main motivations guided the use of multiple small triangles instead of a single, large ROI:

1. Spatial redundancy and local robustness: Different facial subregions respond differently to illumination, specularities, and occlusions (e.g., glasses and hair). Multiple small ROIs provided redundant channels such that the same physiological pulse could appear with different amplitudes and noise realizations across triangles; combining or selecting among these signals increases robustness.
2. Heterogeneous illumination: Under natural lighting (e.g., the COHFACE dataset used here), parts of the face were frequently in shadow. Fine triangulation enabled selective reliance on well-illuminated subregions while discarding shadowed triangles.

### 3.4. Temporally Smoothed Geometric Stabilization

Head rotations introduced rotational components in the image plane and produced apparent scale changes. These motions caused intensity fluctuations unrelated to blood volume dynamics, leading to spurious rPPG artifacts after amplification. To mitigate these motion artifacts, a two-stage geometric stabilization process was implemented, mapping each video frame to a stable, canonical face coordinate system.

Stage 1: Temporally Smoothed Affine Stabilization

A subject-specific canonical reference face was first established by averaging the 2D coordinates of each landmark over the initial n = 15 frames in which a face was reliably detected. For each subsequent frame, an affine transform was estimated between the current landmarks and the canonical reference.

An affine transform provides a compact linear model that maps 2D image coordinates from one frame to another using translation, rotation, uniform scale, and shear terms. In homogeneous coordinates, the 2 × 3 affine transform maps a point $p={\left(x,y\right)}^{T}$ to $p^{\prime }$ as

(1) $$p^{\prime }\;=\;\left[\begin{array}{ccc} a & b & t_{x}\\ c & d & t_{y} \end{array}\right]\left[\begin{array}{c} x\\ y\\ 1 \end{array}\right]\;=\;A\;p+t.$$

If the local deformation is (approximately) a combination of in-plane rotation and isotropic scaling, the linear submatrix can be parameterized as

(2) $$A\;=\;s\left[\begin{array}{cc} \cos\theta & -\sin\theta \\ \sin\theta & \cos\theta \end{array}\right],$$

with translation $t={\left(t_{x},t_{y}\right)}^{T}$. For head rotations, the affine model captures first-order geometric effects that would otherwise generate large, non-physiological pixel intensity variations. This model is appropriate because it: (i) compactly represents rigid head motion (translation, in-plane rotation, and scale); (ii) remains robust to moderate perspective changes; and (iii) is computationally efficient to estimate and invert for image warping.

Direct frame-by-frame application of this transform would have introduced high-frequency jitter due to noise in landmark detection, which could be amplified by EVM. To prevent this, the estimated affine matrix was decomposed into its constituent parameters: translations ($t_{x},t_{y}$); rotation (θ); and scale (*s*). Each parameter $p_{t}$ was then independently smoothed over time using an Exponential Moving Average (EMA):

(3) $${\hat{p}}_{t}=\left(1-\alpha \right)\;{\hat{p}}_{t-1}+\alpha \;p_{t},$$

where ${\hat{p}}_{t}$ was the smoothed parameter at time *t*, and α controlled the trade-off between responsiveness and smoothness. To balance stability against responsiveness to deliberate motion, an adaptive smoothing factor α was used. A low baseline factor ($\alpha_{\mathrm{slow}}=$ 0.05) robustly filtered minor jitter. When a head rotation exceeding 0.05 radians (approximately 2.8 degrees) was detected, a correction phase was initiated in which α was incrementally increased towards a higher value ($\alpha_{\mathrm{fast}}=$ 0.8), allowing the filter to quickly converge on the new head position. Once the rotational error subsided, α returned to $\alpha_{\mathrm{slow}}$ to resume strong smoothing. Finally, a smoothed affine matrix was reconstructed from the adaptively smoothed parameters $\left({\hat{t}}_{x},{\hat{t}}_{y},\hat{\theta },\hat{s}\right)$ and applied to warp the frame.

Stage 2: Translational Stabilization via Optical Flow

After affine stabilization, residual horizontal translation artifacts remained, particularly from side-to-side head movements. To address this, a secondary translational stabilization was applied using Lucas–Kanade OF tracking. Two stable facial landmarks corresponding to the left and right facial boundaries were tracked across consecutive affine-stabilized frames. The mean horizontal displacement of these points relative to their canonical reference positions was computed and used to construct a pure translation matrix, which was then applied to remove residual lateral drift.

**Figure 5:**
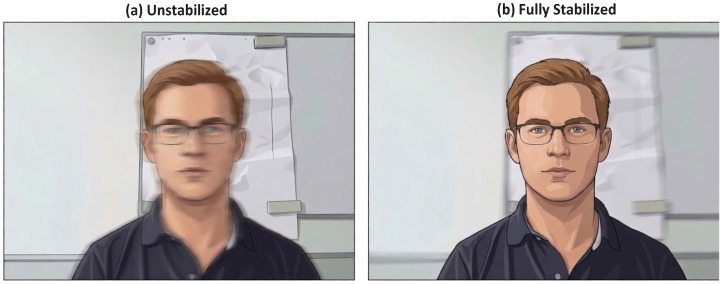
Efficacy of the motion stabilization pipeline. The visual impact of the stabilization algorithm was demonstrated by averaging all frames of a video sequence. (**a**) In the original unstabilized video, subject motion resulted in a blurred temporal mean. (**b**) After applying the full coarse-to-fine stabilization pipeline, frames were aligned to a canonical facial pose, resulting in a sharp, well-defined average image in the facial region, while residual motion blur was relegated to the background. This process was critical for mitigating motion artifacts in the extracted rPPG signal.

This two-stage stabilization method sequentially addressed both large-scale geometric misalignments through the affine transformation and fine-scale translational variations using OF-based correction. To qualitatively assess the effect of this process, Figure 5 presents a visualization obtained by averaging the video sequence into a single representative frame. This averaging was performed solely for visualization purposes and is not part of the stabilization pipeline. The unstabilized video (Figure 5a) appears blurred due to motion, primarily caused by minor head translations. In contrast, the stabilized video (Figure 5b) exhibits a substantial improvement in the quality of the averaged frame, with the facial region clearly defined.

### 3.5. Light-Quality Filtering via Clustering

Although the face detector and triangulation had already localized facial skin, variations in illumination across the face make some triangles unsuitable for rPPG extraction. The pipeline employed an unsupervised clustering step on the per-triangle mean color to identify triangles with chrominance consistent with well-lit skin regions.

**Figure 6:**
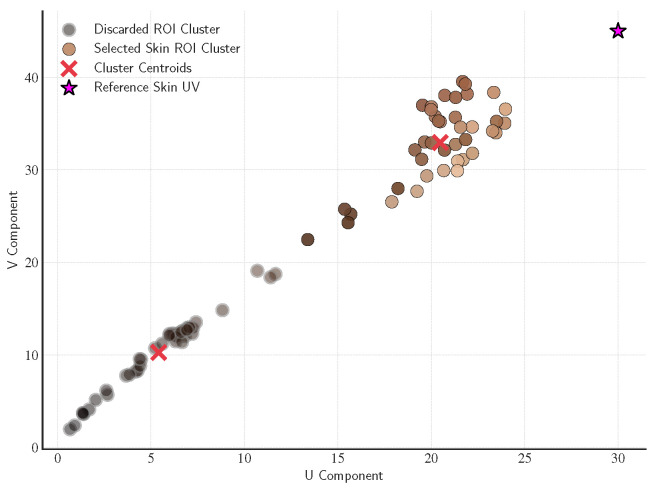
Chrominance-based ROI selection via K-means clustering. Each point color corresponds to the triangle’s temporal mean RGB value, providing a visual cue of its appearance (darker points indicate more poorly illuminated regions, while brighter points correspond to better-lit areas). The plot shows their distribution in the chrominance (U–V) space, where K-means clustering (*k* = 2) separated regions by color similarity. Discarded regions (gray) and the selected ROI cluster are identified, with red crosses marking cluster centroids. The magenta star marks a reference chrominance point empirically representing well-lit regions. The selected cluster effectively isolated well-lit facial regions for rPPG signal extraction.

**Figure 7:**
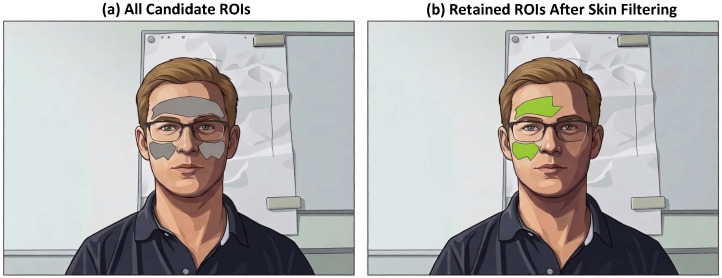
Effect of ROI illumination filtering. This figure illustrated the spatial outcome of the chrominance-based filtering step described in Figure 6. (**a**) All candidate ROIs obtained from the anatomical triangulation are shown. (**b**) Only the ROIs retained after filtering are displayed, corresponding to well-illuminated facial regions suitable for rPPG signal extraction.

The temporal mean RGB for each candidate triangle over the original (non-magnified) video was computed. The mean RGB values were converted to YUV color space, and the chrominance channels (U and V), which are more robust to intensity changes, were retained. K-means clustering with k= 2 was then applied to the UV pairs, and the cluster whose centroid lay nearest to the chrominance range of well-lit regions (empirically set to U ≈ 30 and V ≈ 45) was selected (Figure 6). The exact numerical values are not critical, as the clustering naturally adapts to each scene’s illumination conditions. Triangles in that cluster were retained as the final set of ROIs for signal extraction, while others were discarded (Figure 7). This filtering step substantially improved signal quality by excluding poorly illuminated or occluded regions.

### 3.6. Eulerian Video Magnification (EVM)

The implemented EVM pipeline follows the canonical stages with several optimizations for computational efficiency. Each stabilized frame is decomposed with a Gaussian pyramid and the low-frequency spatial band (pyramid level 3) is selected for temporal analysis to reduce dimensionality and attenuate pixel-level sensor noise.

Temporal filtering (using a bandpass filter with a passband of 0.8–3.0 Hz, corresponding to the typical human heart rate range) and amplification are applied only to pixels within the combined ROI mask derived from skin segmentation, which significantly reduces computation. The filtered low-frequency signal is multiplied by the magnification factor α (set to 50) across all channels (Y, I, and Q). After amplification, the signal is added back to the original YIQ frame, upsampled to the original resolution using cubic interpolation, and converted to RGB.

**Figure 8:**
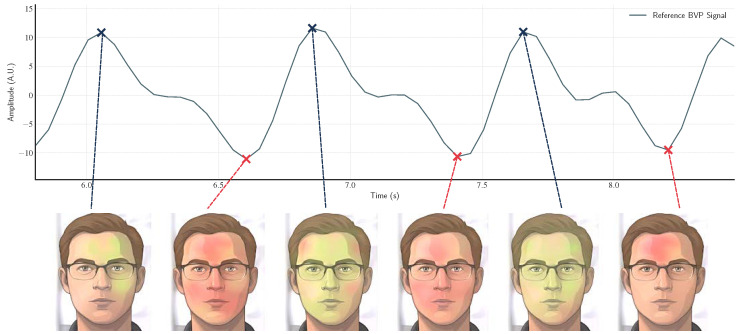
Temporal alignment between the color-amplified video and the reference PPG signal. The color-amplified video is temporally synchronized with the reference PPG waveform. Each peak and trough in the reference signal (marked by x) corresponds to the respective displayed frames below. The color amplification enhances subtle pulsatile variations on the face that coincide with the cardiac cycles in the reference waveform, resulting in alternating dominant green and red hues.

Figure 8 illustrates the temporal correspondence between a magnified facial video and the ground-truth PPG signal. The visual amplification reveals subtle, periodic skin color modulations synchronized with the cardiac cycle, which become discernible at each peak and trough of the reference waveform. It is worth noting the variation, for the same frame, in color intensity across the ROI triangles, with several frames exhibiting mixed or unstable dominant hues. This observation underscores the need for further signal refinement, which is addressed in the subsequent implementation stages.

### 3.7. Saturation Artifact Mitigation

High amplification factors could cause pixels to exceed the valid intensity range [0, 1], producing saturation artifacts that distort the magnified video and degrade downstream signal quality. To mitigate this, the RGB delta $\Delta I=I_{\mathrm{magnified}}-I_{\mathrm{original}}$ was computed for each pixel, and the maximum scaling factor k∈ [0, 1] was determined such that $I_{\mathrm{original}}+k\cdot \Delta I$ remained within [0, 1] for all channels. Specifically, for positive deltas, $k\leq (1-I_{\mathrm{orig}})/\Delta I$; for negative deltas, $k\leq -I_{\mathrm{orig}}/\Delta I$. The minimum *k* across R, G, and B channels was applied to the delta and added to the original RGB, ensuring no channel saturated while maximizing the retained amplification.

The magnified stabilized frames were then clipped to [0, 1] and converted to 8-bit RGB format. For each retained triangle, the mean green channel signal was extracted from the magnified video for subsequent HR estimation. Signals were segmented into 30 s intervals with a 15 s overlap for further processing. This 30 s window provides sufficient temporal information for the subsequent dimensionality reduction and source separation algorithms to converge accurately, while the overlap ensures temporal consistency across the analysis.

### 3.8. Signal Cleaning and Outlier Removal

To mitigate remaining artifacts, such as eye blinks, a signal-level outlier removal procedure was applied.

A modified outlier score was computed for each signal using the Median Absolute Deviation (MAD). For a signal x(t), the modified z-score was defined as

(4) $$z_{\mathrm{mod},i}=0.6745\cdot \frac{x_{i}-\mathrm{median}\left(x\right)}{\mathrm{MAD}(x)},$$

where MAD(x)=median(|x−median(x)|). Points satisfying $|z_{\mathrm{mod},i}|>$ 3 were classified as outliers. An outlier ratio was computed as the number of outliers divided by the sum of outliers and overall detected peaks. Signals with an outlier ratio exceeding 0.4 were discarded, as they were dominated by non-physiological artifacts.

### 3.9. Harmonic Suppression

A well-known challenge in rPPG analysis was the presence of harmonics, i.e., spectral peaks at integer multiples of the true HR frequency. In particular, the first harmonic ($2f_{0}$) often appeared stronger than the fundamental, potentially leading to incorrect rate estimation. To address this, a harmonic suppression strategy was applied.

The dominant frequency was first detected in the power spectrum by identifying the peak magnitude. This peak was then checked to determine if it corresponded to a harmonic by inspecting the neighborhood of the half-frequency (±1 FFT bin) for significant energy (>40% of the peak magnitude). If a fundamental candidate was found, it was selected; otherwise, the original peak was retained.

Once the fundamental $f_{0}$ was identified, an adaptive Gaussian notch filter was applied at the first harmonic location $2f_{0}$:

(5) $$H\left(f\right)=1-\alpha \exp\left(-\frac{{\left(f-2f_{0}\right)}^{2}}{2\sigma^{2}}\right),$$

where σ=0.1 Hz controls the bandwidth and α= 0.7 the attenuation depth. The spectral energy removed from the harmonic was redistributed to the fundamental bin to preserve overall signal energy, computed by adding the removed complex amplitude in-phase with the fundamental: $\mathrm{FFT}\left(f_{0}\right)\leftarrow \mathrm{FFT}\left(f_{0}\right)+\Delta E\cdot e^{i\phi_{0}}$, where $\phi_{0}$ was the phase of the fundamental. This process enhanced the physiological interpretability of the rPPG signals by enforcing dominance of the true cardiac rhythm.

### 3.10. Sliding-Window PCA for Local Coherence

Physiological oscillations exhibited spatial coherence, although their amplitude and Signal-to-Noise Ratio (SNR) varied across facial regions due to lighting conditions, pose, and skin characteristics. To capture locally coherent dynamics while preventing dominance by high-amplitude traces, a sliding-window PCA was applied to the set of signals.

A window was moved across the signal index with a fixed stride. The window size was chosen adaptively as windowsize=min(max(M/3;5);M), where *M* denoted the number of filtered traces, and the stride was fixed to three traces. This procedure generated overlapping groups that (i) captured local modes of coherence, (ii) reduced sensitivity to artifacts from individual ROIs, and (iii) leveraged spatial and illumination heterogeneity by allowing multiple overlapping groups to represent the same large-scale pulsatile mode. For each group, PCA was computed with two PCs.

**Figure 9:**
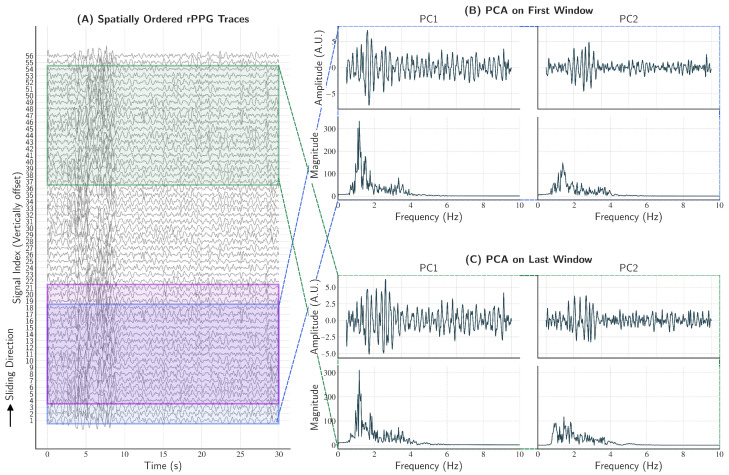
Visualization of the sliding-window PCA method for extracting locally coherent signals. This diagram illustrates how PCA is applied to spatially adjacent groups of signals to isolate the dominant pulsatile component. (**A**) The input matrix of *M* rPPG signals is shown, vertically offset for clarity. Overlapping windows are applied to group signals based on their spatial proximity, with three such windows highlighted. The first two consecutive windows demonstrate the overlap and stride of the process (blue and purple), while the third window (green) illustrates a later strided position along the signals set. (**B**,**C**) A detailed “zoom-in” on the PCA results for two spatially distant windows (the first and the last) as indicated by the dashed callout lines. For each window, a 2 × 2 grid displays the first two PCs (PC1 and PC2) in both the time and frequency domains. In this example the first PC (PC1) in both cases successfully captures a strong, narrow-band peak within the cardiac frequency range.

Figure 9 illustrates the concept and outcome of the sliding-window PCA approach.

### 3.11. Correlation-Based Component Selection

To further enhance physiological consistency before global dimensionality reduction, the pairwise correlation matrix of the grouped components was computed. For each component, the mean absolute correlation with all others was evaluated to identify the most representative signal. Components whose absolute correlation with this representative exceeded a threshold of 0.5 were retained. This correlation-based filtering step effectively preserved signals exhibiting mutual coherence, discarding spurious components influenced by local noise or motion.

### 3.12. Global PCA and SOBI for Source Separation

The remaining components were then reduced through a second, global PCA, restricted to a maximum of ten components. This global step (i) concentrated the signal energy into a small set of orthogonal modes for denoising and (ii) performed dimensionality reduction, constraining the subsequent Blind Source Separation (BSS) to a compact and computationally efficient subspace.

SOBI was then applied to the reduced set of components to separate the underlying sources based on second-order temporal statistics. SOBI performs joint diagonalization of time-delayed covariance matrices, making it particularly effective for isolating structured, quasi-periodic signals such as cardiac pulsations from noise. The output was a set of independent candidate sources, among which the rPPG component was expected to emerge.

Before feature extraction, harmonic suppression (as described previously) and time point-level outlier removal were applied to each SOBI component to enhance spectral clarity. Outliers time points were identified by comparing each signal x(t) to its mean μ and standard deviation σ. A point $x_{i}$ was classified as an outlier if

(6) $$|x_{i}-\mu |>\tau \sigma ,$$

with threshold τ= 4. Such values were replaced with zeros, and the resulting signal was subsequently bandpass filtered to mitigate potential discontinuities introduced by this zero replacement.

### 3.13. Feature Extraction and Source Selection

To identify the SOBI components corresponding to physiological sources, a supervised machine learning approach was employed. A dataset was constructed containing all sources generated during the procedure for the training subset videos. Each source within the training subset was manually annotated as class 1 (pulse signal) by comparing its waveform and peak Fast Fourier Transform (FFT) frequency with the reference signal. All other sources were labeled as class 0. It is important to emphasize that this manual labeling was utilized strictly to establish the initial training subset and does not impact the objectivity of the testing phase, nor is it required during future inference, thereby preserving the pipeline’s scalability.

Following this labeling, the dataset consisted of 4638 sources, of which 4030 were class 0 and 608 were class 1.

For each component, wavelet scattering features were extracted from both the signal and its autocorrelation function to capture temporal and spectral structure.

**Figure 10:**
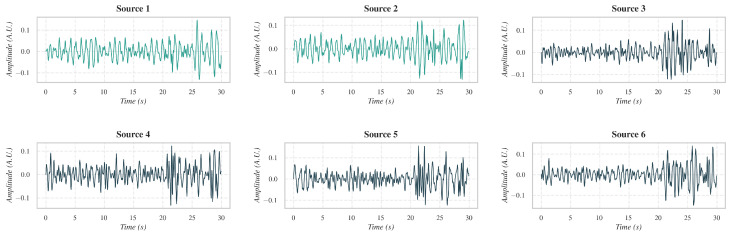
Visualization of SOBI components after source selection. The components highlighted in green correspond to valid physiological signals, as identified by the feature-based LightGBM classifier. The remaining components (in dark blue) were classified as non-physiological or noise. Only the first six of the ten SOBI components are displayed; the remaining components corresponded to noise sources.

A Light Gradient Boosting Machine (LightGBM) classifier was trained on the training subset of the COHFACE dataset using Leave-One-Subject-Out Cross Validation (LOSOCV) to prevent overfitting. For each test video, the classifier was retrained excluding that video’s data. The classifier outputted the probability of each component representing a valid rPPG source. Components with probability exceeding a decision threshold of 0.35 were retained for final reconstruction (Figure 10). It should be noted that while LOSOCV requires model retraining for each subject during this validation phase, a real-world deployment would utilize a single, fixed pre-trained model, thereby introducing no per-subject retraining overhead.

If no components exceeded the classification threshold, a fallback mechanism was employed. The variance explained by grouped PCs was inspected: if the first SOBI component accounted for >75% of total signal power (as measured by the component power ratio), it was selected as the sole physiological source. This 75% threshold was determined empirically based solely on observations from the training subset, where true physiological components consistently dominated the signal variance when successfully separated. This fallback ensured robustness when the classification model was overly conservative.

### 3.14. Final Signal Reconstruction and Rate Estimation

The selected components were back-projected through inverse transformation to reconstruct denoised signals in the original signal space. Temporal alignment was then applied to correct phase shifts across sources by maximizing cross-correlation within the physiological frequency band. This ensured that inter-ROI delays did not attenuate the pulsatile oscillation during averaging. The aligned signals were averaged to produce a single representative trace for the current episode.

To enhance short-term rate estimation while preserving temporal continuity across episodes, a segment-linking procedure was applied. The final 15 s of the current episode were compared with the final 15 s of the previous episode. When both segments contained valid (non-null) sources, PCA was applied to the concatenated pair, and the first PC (capturing the mode of highest explained variance) served as the representative trace for rate estimation. If one segment was null, the non-null segment was used directly. This approach mitigated boundary effects and improved robustness to transient artifacts.

**Figure 11:**
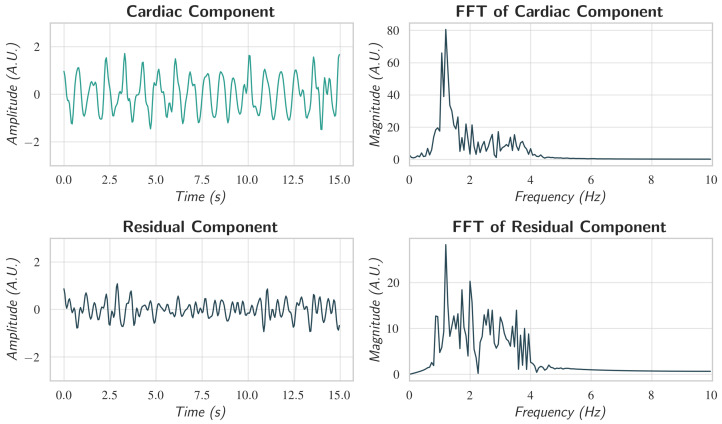
Cardiac and residual components after segment linking. The upper panels show the representative cardiac component obtained through the segment-linking procedure, and its corresponding frequency spectrum, where a dominant peak is observed at the cardiac frequency. The lower panels present the residual component and its spectrum, illustrating the suppression of physiological oscillations after component separation.

Finally, the HR was estimated by computing the FFT of the representative 15 s trace, identifying the dominant spectral peak, and multiplying its frequency by 60 (Figure 11).

### 3.15. Evaluation

For each experiment, agreement with reference measurements was evaluated using Bland–Altman plot analysis. The *x*-axis represents the mean of the two measurements, and the *y*-axis shows their difference. The plot includes a horizontal line indicating the bias, computed as the average difference between methods, and the Limits of Agreement (LoA), defined as the mean difference ±1.96 standard deviations. The goal is to achieve low bias and narrow limits of agreement.

To facilitate comparison with existing literature, additional metrics were computed: Mean Absolute Error (MAE), Root Mean Square Error (RMSE), Mean Error Rate (MER), and Pearson Correlation Coefficient (PCC).

## 4. Results

This section presents the performance of the proposed contactless HR estimation framework. The evaluation includes the performance of the classifier for source selection, the overall performance of the full approach on various subsets of the COHFACE dataset, and a comprehensive ablation study to quantify the contribution of each component within the proposed pipeline.

### 4.1. Source Selection Classifier Performance

The performance of the LightGBM classifier for selecting the correct physiological source was evaluated on the test set. As detailed in Table 2, the classifier achieved a high F1-score of 0.839, demonstrating a strong balance between precision (0.870) and recall (0.810). The overall accuracy was 0.961, indicating a high rate of correct classification for both physiological and non-physiological components.

### 4.2. Performance on the COHFACE Dataset

The proposed approach was evaluated on the COHFACE dataset, analyzing its performance on the complete dataset, on subsets with studio and natural lighting, and on the official test set partition. The results of this evaluation are presented in Table 3.

On the official test set (n = 64), the proposed method achieved an MAE of 1.50 bpm, an RMSE of 3.07 bpm, and a Mean Error Rate (MER) of 2.46%. A high PCC of 0.97 was observed between the estimated and ground truth HRs. The performance remained robust across different lighting conditions, with an MAE of 1.97 bpm under studio lighting compared to 2.21 bpm under natural lighting. However, a non-parametric Mann–Whitney U test on the absolute errors between the two conditions (a test choice justified by the non-normal distribution of absolute errors) found that this difference was not statistically significant (*p* = 0.583), suggesting the proposed method is equally effective in both lighting environments.

**Figure 12:**
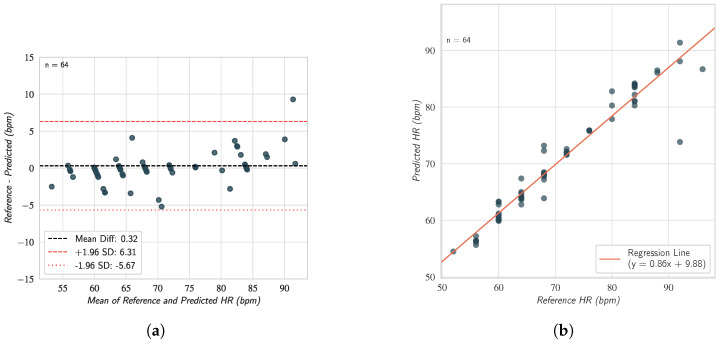
Comparison between the reference values and the corresponding estimates according to the (**a**) Bland–Altman and (**b**) correlation plots for the HR estimation on the COHFACE test set (n = 64). Each dot represents a single paired reference–estimate measurement (one sample from the test set).

The agreement and correlation between the reference and predicted HRs on the test set are visualized in Figure 12. The Bland–Altman plot (Figure 12a) shows a mean difference (bias) of 0.32 bpm, with 95% limits of agreement (LoA) between −5.67 bpm and 6.31 bpm. The correlation plot (Figure 12b) further confirms a strong linear relationship (r = 0.97) between the ground truth and the estimated values.

**Figure 13:**
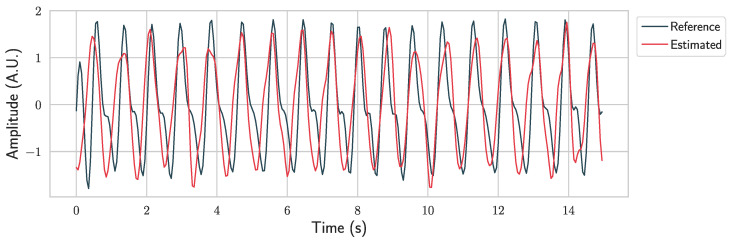
Example of the reference and estimated pulse signals over a 15 s segment from the COHFACE dataset. The estimated signal closely follows the temporal variations of the reference, demonstrating the ability of the proposed method to accurately capture HR dynamics.

Figure 13 shows the overlay between the reference and estimated HR signals for a representative subject, demonstrating the temporal and amplitude agreement between the two.

To comprehensively situate the performance of the proposed framework, it was benchmarked against existing methods on the COHFACE dataset.

**Figure 14:**
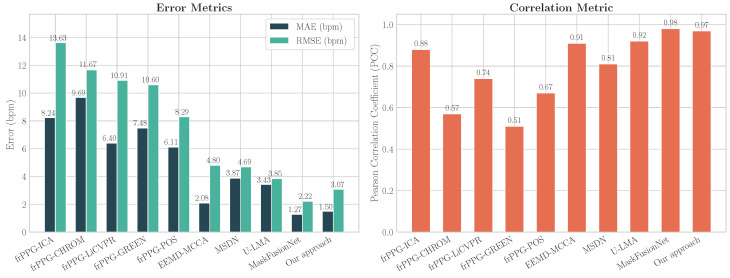
Performance comparison of HR estimation methods using the COHFACE testing dataset. The left panel presents the error metrics (MAE and RMSE) for each method, where lower values are better. The right panel shows the correlation metric (PCC) between the predicted and reference HR values, where higher values are better.

Table 4 and Figure 14 present this detailed comparison. To provide a robust and complete baseline for traditional algorithms, all five optimized pipelines from the flexible framework (frPPG) by Dawoodjee and Ghahramani [26] are included.

As the results demonstrate, the proposed approach achieves a substantial performance improvement over all compared optimized traditional methods and most recent SOTA methods. It obtains a MAE of 1.50 bpm and RMSE of 3.07 bpm. While MaskFusionNet achieves a lower MAE of 1.27 bpm and RMSE of 2.22 bpm, our method maintains a high PCC of 0.97, indicating strong linear agreement between the estimated and ground truth HRs, and surpasses all other reported methods.

Furthermore, to assess the framework’s robustness under different environmental conditions, performance was evaluated separately on the studio and natural lighting subsets of the COHFACE dataset. Table 5 compares the proposed approach with methods that have also reported results under these specific conditions.

Under studio lighting, the proposed approach achieved an MAE of 1.97 bpm and an RMSE of 5.01 bpm. Under the more challenging natural lighting conditions, the method maintained strong performance with an MAE of 2.21 bpm and an RMSE of 4.74 bpm.

### 4.3. Ablation Study

An ablation study was conducted on the test set to evaluate the impact of removing individual components from the proposed pipeline. The following model variations were evaluated: without stabilization; without clustering; without EVM; without Sliding-Window PCA; and without any postprocessing, i.e., including only the processing up to the extraction of the mean green channel (Section 3.7) and computing the FFT peak directly from the average of the triangle time-series signals. The results, presented in Table 6, demonstrate that the full approach achieves the best performance across all metrics. Removing any component led to a degradation in performance, highlighting the contribution of each stage to the final result.

**Figure 15:**
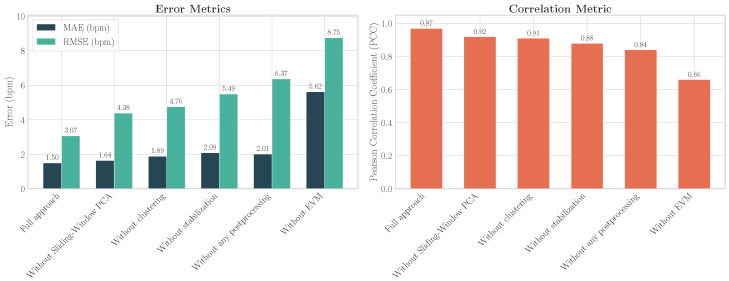
HR ablation study performance comparison on the COHFACE test set. The left panel shows error metrics (MAE and RMSE), where lower values are better. The right panel shows the correlation metric (PCC), where higher values are better. Ablations are sorted by increasing RMSE to illustrate the performance degradation.

The most critical component was identified as the EVM step. Its removal resulted in the most significant performance drop, with the MAE increasing to 5.62 bpm, the RMSE rising to 8.75 bpm, and the PCC falling to 0.66. Furthermore, this was the only configuration that did not achieve a 100% detection rate on the test set, succeeding on only 79.7% of the test videos. The performance degradation of each ablated model is visualized in Figure 15.

**Figure 16:**
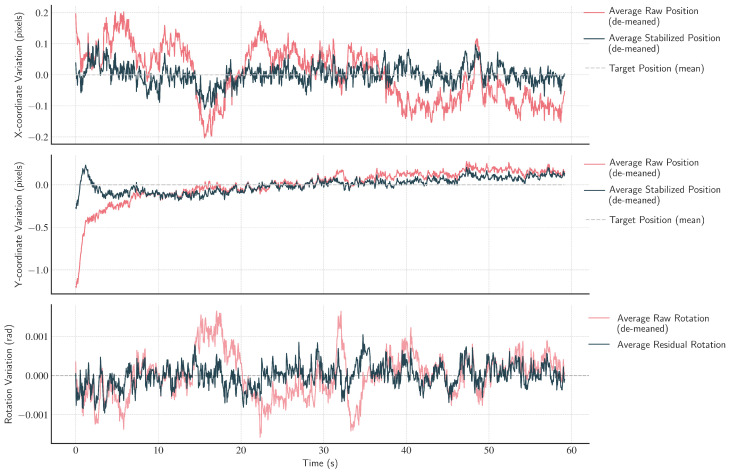
Average quantitative analysis of stabilization performance across the dataset. The plots illustrate the mean effect of the stabilization algorithm, averaged over all videos. They track the position of a cheek landmark and the overall head rotation over time. The signals are de-meaned for each video before averaging to show variation rather than absolute position. (**Top**) and (**Middle**) display the X and Y coordinate variations, respectively. The raw, pre-filtered input motion (red) shows significant low-frequency drift, which is effectively corrected in the stabilized output (dark blue), keeping it centered around the target position (dashed line). (**Bottom**) displays head rotation, where the raw input rotation (red) is substantially dampened, resulting in a residual rotation (dark blue) with a much smaller amplitude.

The effectiveness of the stabilization pipeline was quantitatively evaluated across the entire dataset, with results averaged to demonstrate overall performance (see Figure 16). Positional and rotational jitter were calculated during high-motion segments (inter-frame velocity exceeding μ+1σ). Positional jitter was defined as the spatial standard deviation ($\sqrt{\sigma_{x}^{2}+\sigma_{y}^{2}}$) of a cheek landmark, while rotational jitter was the standard deviation of the affine rotation angle. The analysis confirmed that the proposed method significantly reduced both low-frequency drifts and high-frequency jitter in facial motion. As shown in Table 7, the average positional jitter decreased by approximately 40%, from 1.41 pixels to 0.85 pixels. Rotational jitter was reduced by 50%, and its variance across videos decreased by over 70%, indicating consistent stabilization performance across subjects.

## 5. Discussion

The primary objective of this work was to develop a robust, contactless framework for HR estimation designed to address persistent challenges in rPPG measurement, namely motion artifacts, variable illumination, and low SNR. The results presented in Section 4 demonstrate that the proposed multi-stage, hybrid pipeline fulfills this objective, delivering SOTA accuracy on the challenging COHFACE dataset.

The framework achieved a MAE of 1.50 bpm, a RMSE of 3.07 bpm, and a PCC of 0.97 on the COHFACE test set (Table 3). This performance is notable given the significant compression artifacts and challenging illumination conditions inherent to this dataset. Furthermore, the pipeline demonstrated resilience to lighting variations, with no statistically significant difference in performance between the “Studio” (MAE 1.97 bpm) and “Natural” (MAE 2.21 bpm) subsets.

It should be noted that of the 160 videos in the dataset, 159 were successfully processed. In one instance, neither the classifier nor the fallback threshold could isolate a viable physiological component, resulting in a safely yielded null result rather than a spurious estimation. While this specific video featured a subject with a darker skin tone under low natural lighting, the pipeline successfully processed a second video from the exact same participant under identical conditions. Consequently, this null output is attributed to a transient drop in the Signal-to-Noise Ratio rather than a fundamental demographic failure. In real-world clinical applications, which rely on continuous monitoring rather than isolated 30 s segments, a transient null output acts as a safely dropped data point. This conservative, fail-safe mode is highly preferable to reporting an erroneous measurement, which could otherwise trigger false clinical alarms or mask genuine physiological events.

The following discussion positions the framework within the current SOTA, details the architectural motivation, and examines the contribution of each module through ablation analysis.

### 5.1. Comparison with the State of the Art

Table 4 presents a direct comparison of the proposed framework with traditional and deep learning SOTA methods. The achieved performance (RMSE = 3.07 bpm) is substantially lower than that of other traditional and hybrid approaches, including EEMD-MCCA [18] (4.80 bpm) and U-LMA [13] (3.85 bpm). The framework yielded a 34% reduction in RMSE relative to the complex MSDN architecture [21] (4.69 bpm), whose reliance on pseudolabels from traditional algorithms (e.g., CHROM and POS) constrains its performance. In contrast, the proposed model identifies valid physiological components through supervised classification of wavelet-based features rather than reproducing the output of a specific algorithm.

The very recent MaskFusionNet [23] reported an MAE of 1.27 bpm, slightly surpassing the 1.50 bpm obtained in this work. While this highlights the potential of masked auto-encoding and transformer-based architectures, these models require substantial pre-training and exhibit high computational complexity. The contribution of the present framework lies in reaching competitive performance with an interpretable hybrid design in which each processing stage can be explicitly validated (Figure 5, Figure 6 and Figure 10). This transparency is advantageous for clinical and other high-stakes applications where model failure modes must remain observable [14].

### 5.2. Rationale for a Multi-Stage Signal Processing Pipeline

A core premise of this work is that robust rPPG estimation is unlikely to be achieved through a single algorithmic mechanism. The rPPG signal is intrinsically weak and easily obscured by motion, illumination changes, and compression artifacts. The proposed framework therefore adopts a sequential mitigation strategy in which each processing stage is designed to attenuate a specific source of noise, progressively isolating and refining the underlying physiological signal.

This system-level perspective is supported by the findings of Dawoodjee and Ghahramani [26], who highlighted that benchmark algorithm performance depends critically on the surrounding processing pipeline. They also reported substantial variability across the literature due to inconsistent evaluation procedures, making direct and accurate comparison between methods difficult. The multi-stage design used in this work addresses these concerns by enforcing a consistent, structured progression from raw video to cleaned physiological traces.

### 5.3. Interpretation of the Ablation Study Architecture

The ablation study (Table 6) quantitatively validates the contribution of each component. Removing the two-stage stabilization module led to a significant degradation (RMSE 5.49 bpm), showing that motion compensation remains essential even in datasets with limited movement. The temporally smoothed affine correction (Stage 1) and OF-based refinement (Stage 2) reduced spatial jitter and improved alignment (Figure 5 and Figure 16, Table 7).

Eliminating the illumination-robust clustering stage increased error (RMSE 4.76 bpm), confirming the value of the multi-patch tessellation and chrominance-based filtering for handling heterogeneous lighting. This contrasts with HRMSF [15], which reported substantially higher errors despite also using multi-patch sampling.

The removal of EVM caused the largest decline (RMSE 8.75 bpm) and frequent signal-extraction failures, indicating that amplification is critical under severe compression. COHFACE’s very low bitrate (0.25 Mb/s) makes raw rPPG traces nearly indistinguishable from quantization noise, and EVM restores detectable physiological variability.

Finally, the ablation of the signal-separation and selection stages highlights the importance of expanding dimensionality before decomposition and replacing heuristic component selection with a supervised classifier. The combination of dense triangulation, sliding-window PCA, SOBI, and LightGBM classification achieved superior robustness, with RMSE increasing to 4.38 bpm when PCA was removed and an F1-score of 0.839 (Table 2). Although multiple components were occasionally labeled as physiological during annotation, identifying at least one valid source was sufficient for reconstructing a clean pulse signal.

### 5.4. Data Minimization and Privacy Considerations

The heart rate framework proposed in this work implements a proactive data minimization strategy designed to protect user data. While any system that even temporarily processes raw facial video inherently encounters privacy considerations, our approach relies on immediate abstraction to prevent data exposure. At no point does the methodology require or allow the long-term storage of identifiable facial video; raw frames are discarded immediately after initial processing. Furthermore, the pipeline selectively targets only the forehead and cheek ROIs, excluding regions with high biometric content such as the eyes and mouth. During signal extraction, the red and blue color channels are discarded, preserving only the amplified green-channel time series (a non-identifiable representation of skin chrominance fluctuation). Because this downstream signal cannot be reverse engineered or reconstructed into a facial image, the framework provides highly robust protection against the long-term exposure of personal biometric information through strict data minimization.

### 5.5. Computational Complexity

Regarding computational complexity and real-time feasibility, the processing time was evaluated across 25 trials conducted on the COHFACE dataset using a standard consumer laptop (AMD Ryzen 7 6800HS, 16 GB RAM, without dedicated GPU acceleration), and consists of two main phases. The first phase, comprising facial tracking, stabilization, and EVM, operates at approximately 20 FPS. The second phase, which encompasses the time-series signal extraction, sliding-window PCA, SOBI, LightGBM classification, and final HR estimation, requires approximately 0.41 s to fully process a 30 s segment. Given that the video processing phase closely approaches standard camera acquisition rates purely on CPU architecture, and the signal processing phase resolves in two orders of magnitude lower than the data window size, the framework demonstrates strong potential for integration into real-time continuous monitoring systems with minor further software optimization.

## 6. Conclusions

In conclusion, this work successfully designed, implemented, and validated a novel and robust framework for contactless heart rate monitoring. By systematically addressing the challenges of motion, illumination, and Signal-to-Noise Ratio with dedicated, interpretable processing stages, this work demonstrates the potential of a hybrid signal processing and machine learning paradigm.

Our approach combines geometric stabilization, dense facial triangulation, unsupervised ROI filtering, EVM, sliding-window PCA, and a SOBI–LightGBM source selection module. Validation on the COHFACE dataset showed highly competitive performance, with an overall MAE of 1.50 bpm and a PCC of 0.97. This accuracy approaches that of complex end-to-end deep learning models (e.g., MaskFusionNet) and significantly outperforms other traditional and hybrid SOTA methods (e.g., MSDN and U-LMA). The method also exhibits robustness to video compression and variable illumination.

While requiring facial data, the approach implements strict data minimization techniques: it processes only triangular ROIs from the cheeks and forehead, excludes high-identity regions (e.g., eyes), and immediately converts identifiable video into abstract, non-reconstructible time-series data for analysis.

Despite the strong validation results, this work presents limitations that inform directions for future research.

Methodologically, the reliance on the Fast Fourier Transform (FFT) for average rate estimation was chosen for its robustness against motion artifacts and independence from perfect peak morphology. However, in future work, this could be extended using time-domain peak detection or time-frequency analysis, such as the Continuous Wavelet Transform (CWT), to enable the assessment of Heart Rate Variability (HRV).

The harmonic suppression step, hypothesized to improve robustness against spectral misidentification (i.e., $f_{0}$ vs $2f_{0}$), was not extensively validated. The COHFACE dataset presented few clear instances of harmonic dominance, limiting the ability to quantify this module’s impact. In continuous, real-world monitoring, where signal quality varies, harmonics may be more frequent. Future studies should evaluate this module on datasets with pronounced harmonic artifacts. Additionally, the LightGBM classifier successfully enables robust source selection without manual intervention during inference. The current training set was established through careful manual annotation to ensure high data quality. To facilitate seamless scaling of the training set in future work, the annotation process could be automated by calculating the Pearson correlation between the separated sources and reference signals, further streamlining the pipeline. Finally, a limitation of the current study is the unassessed potential for demographic bias in the HR pipeline. Validation was performed on the COHFACE dataset, which, like many public rPPG datasets, predominantly includes individuals with lighter skin tones [27]. This is critical, as rPPG is sensitive to skin tone: higher melanin concentrations absorb more light, reducing signal intensity and increasing estimation errors [27]. Therefore, the robustness of the proposed HR pipeline should be evaluated on more ethnically diverse datasets, such as MMSE-HR or BP4D+, to ensure equitable applicability.

This research provides a solid technological foundation for developing unobtrusive, continuous vital sign monitoring systems, moving the field closer to deploying reliable, camera-based monitoring in clinical and home environments, ultimately enhancing patient safety, comfort, and proactive healthcare.

## Figures and Tables

**Table 1:** Summary of related work on heart rate (HR) estimation through video.

Study	Objective	Innovation/Methods	Relevant Results	Main Limitations
Qi et al., 2017 [16]	Improve HR estimation via correlations across facial sub-regions using J-BSS	Sub-region definition via facial landmarks; C-MCCA learns optimal connectivity for BVP recovery	DEAP: RMSE = 5.00 bpm, PCC = 0.74; outperforms ICA methods	Heuristic spectral-peak source selection; computationally intensive training
Gupta et al., 2022 [13]	Motion- and illumination-robust rPPG extraction using undercomplete ICA	U-LMA: undercomplete ICA with customized Levenberg–Marquardt optimization	COHFACE: RMSE = 3.85 bpm, PCC = 0.92	Periodic-signal fusion hampers BVP–noise separation; sensitive to local artifacts
Boccignone et al., 2025 [14]	Enhance robustness via selection of reliable signals from facial patches	Patch-based pipeline with CIRCLECLUSTERING for PSD-based pulsatile-signal selection	Across five datasets, average MAE = 1.38 bpm (PURE, UBFC)	Dependent on base rPPG method; patch clustering adds computational cost
Chen et al., 2024 [15]	Improve robustness to motion and illumination variations	Multipatch Spatiotemporal Features; CHROM-EGC with adaptive green-component correction	PURE: MAE = 0.96 bpm, RMSE = 1.48 bpm; degraded on COHFACE (MAE = 10.34 bpm)	Non-adaptive illumination correction; reduced performance at low frame rates
Das et al., 2022 [17]	Mitigate motion and illumination artifacts before rPPG extraction	2D Variational Mode Decomposition (2D-VMD) with AAPSD and kurtosis-based mode selection	COHFACE: RMSE decreased from 16.89 bpm to 2.41 bpm; PCC improved to 0.98	Excludes head-rotation cases; mode selection relies on fixed heuristics
Song et al., 2021 [18]	Handle uneven illumination in rPPG estimation	EEMD decomposition of patch signals; MCCA for correlated-component extraction	COHFACE: RMSE = 4.80 bpm, PCC = 0.91	Hard ROI selection limits diversity; EEMD amplifies in-band noise; high computational cost
Chen and McDuff, 2018 [19]	First end-to-end deep model (DeepPhys) for HR and BR estimation	Two-stream Convolutional Attention Network (appearance attention + motion difference input)	MAHNOB-HCI: MAE = 4.57 bpm	Limited interpretability; attention highlights but does not isolate BVP
Castellano Ontiveros et al., 2024 [20]	Reconstruct high-quality rPPG via supervised learning	LSTM network trained on outputs of ICA, CHROM, LGI, POS methods	PURE: generalizes to unseen datasets with PCC ≈ 0.8	Sensitive to ROI averaging; black-box model may suppress subtle features; computationally heavy
Zhang et al., 2023 [21]	Develop a multistage deep model for robust HR estimation	MSDN: HR-aware feature extraction, SVD-based rPPG generation, IBI-based HR estimation	COHFACE: MAE = 3.87 bpm; strong cross-dataset performance	Pseudolabel biases propagate; IBI stage requires clean waveforms
Yu et al., 2022 [22]	Leverage transformers for long-range temporal modeling	PhysFormer: temporal-difference convolutions with self-attention and curriculum frequency loss	VIPL-HR: MAE = 4.97 bpm; cross-dataset MAE = 2.84 bpm on MMSE-HR	Complex training strategy; high computational cost
Zhang et al., 2024 [23]	Improve generalization via masked pre-training across facial regions	MaskFusionNet: dual-stream transformer with masked autoencoder pre-training and temporal fusion	VIPL-HR: MAE = 4.37 bpm; COHFACE: MAE = 1.27 bpm; strong cross-dataset performance	High computational cost; large data requirements

*Abbreviations:* 2D-VMD: 2D Variational Mode Decomposition; AAPSD: Azimuthally Averaged Power Spectrum Density; BR: Breathing Rate; BVP: Blood Volume Pulse; C-MCCA: Connectivity Multiset Canonical Correlation Analysis; CHROM: Chrominance; EEMD: Ensemble Empirical Mode Decomposition; EGC: Extra Green Component; IBI: Interbeat Interval; J-BSS: Joint Blind Source Separation; LGI: Local Group Invariance; LSTM: Long Short-Term Memory; MCCA: Multiset Canonical Correlation Analysis; MSDN: Multistage Deep Network; POS: Plane-Orthogonal-to-Skin; PSD: Power Spectral Density; SVD: Singular Value Decomposition; U-LMA: Undercomplete Levenberg-Marquardt Algorithm.

**Table 2:** The HR classification performance for the source selection using a LightGBM classifier on the COHFACE test set.

Model	Accuracy	Precision	Recall	F1-Score
Proposed approach	0.961	0.870	0.810	0.839

**Table 3:** The HR performance results on the COHFACE dataset across different subsets. n is the number of successfully processed videos in each subset.

COHFACE Subset	n	MAE (bpm)	RMSE (bpm)	MER (%)	PCC	SD (bpm)	Mean Bias (bpm)
All videos	159	2.09	4.88	3.31	0.91	4.88	0.04
Studio Lighting	80	1.97	5.01	3.25	0.91	4.99	0.48
Natural Lighting	79	2.21	4.74	3.37	0.92	4.72	−0.40
Test set	64	1.50	3.07	2.46	0.97	3.06	0.32

**Table 4:** Comparison of the HR results between existing methods and our approach using the testing data provided by COHFACE dataset (n = 64). Compared values are sourced from the literature to provide a contextual performance baseline.

Methods	MAE (bpm)	RMSE (bpm)	PCC	SD (bpm)
Optimized Traditional Methods [26]				
frPPG-ICA	8.24	13.63	0.88	–
frPPG-CHROM	9.69	11.67	0.57	–
frPPG-LiCVPR	6.40	10.91	0.74	–
frPPG-GREEN	7.48	10.60	0.51	–
frPPG-POS	6.11	8.29	0.67	–
Recent SOTA Methods				
EEMD-MCCA [18]	2.08	4.80	0.91	4.33
MSDN [21]	3.87	4.69	0.81	4.19
U-LMA [13]	3.43	3.85	0.92	3.86
MaskFusionNet [23]	1.27	2.22	0.98	–
Our approach	1.50	3.07	0.97	3.06

The MAE for U-LMA was not reported in [13] and was approximated from the mean error and standard deviation, assuming Gaussian-distributed errors: $\mathrm{MAE}\approx \left|\mathrm{mean}\_\mathrm{error}\right|+\mathrm{SD}\cdot \sqrt{2/\pi }$.

**Table 5:** Comparison of the HR results between existing methods and our approach under different lighting conditions (studio and natural lighting) on the COHFACE dataset. Compared values are sourced from the literature to provide a contextual performance baseline.

Scenario	Method	MAE (bpm)	RMSE (bpm)	PCC	SD (bpm)
Studio lighting	HRMSF [15]	10.34	12.04	–	5.87
	2D-VMD [17]	2.72	2.41	0.98	2.23
	EEMD-MCCA [18]	1.41	3.26	0.96	2.94
	Our approach	1.97	5.01	0.91	4.99
Natural lighting	HRMSF [15]	13.41	15.07	–	6.71
	2D-VMD [17]	2.48	2.51	0.99	2.47
	EEMD-MCCA [18]	2.79	6.03	0.85	5.34
	Our approach	2.21	4.74	0.92	4.72

The MAE for 2D-VMD was not reported in [17] and was approximated from the mean error and standard deviation, assuming Gaussian-distributed errors: $\mathrm{MAE}\approx \left|\mathrm{mean}\_\mathrm{error}\right|+\mathrm{SD}\cdot \sqrt{2/\pi }$.

**Table 6:** Ablation study results on the COHFACE test set (n = 64), showing the impact of processing step removal from the HR estimation pipeline. Models are sorted by ascending RMSE to show the trend of performance degradation.

Ablation	MAE (bpm)	RMSE (bpm)	PCC	SD (bpm)	Mean Bias (bpm)
Full approach	1.50	3.07	0.97	3.06	0.32
Without Sliding-Window PCA	1.64	4.38	0.92	4.36	−0.34
Without clustering	1.89	4.76	0.91	4.71	−0.65
Without stabilization	2.09	5.49	0.88	5.44	−0.71
Without any postprocessing	2.01	6.37	0.84	6.24	−1.26
Without EVM	5.62	8.75	0.66	8.75	−0.32

**Table 7:** Quantitative stabilization metrics (mean ± SD).

Metric	Before Stabilization	After Stabilization
Positional Jitter (pixels)	1.405±0.866	0.846±0.284
Rotational Jitter (radians)	0.011±0.012	0.005±0.003

## Data Availability

The research in this paper used the COHFACE Dataset made available by the Idiap Research Institute, Martigny, Switzerland.

## Abbreviations

The following abbreviations are used in this manuscript:

bpm	beats per minute
BSS	Blind Source Separation
CWT	Continuous Wavelet Transform
EMA	Exponential Moving Average
EVM	Eulerian Video Magnification
FFT	Fast Fourier Transform
FPS	Frames per Second
HR	Heart Rate
HRV	Heart Rate Variability
ICA	Independent Component Analysis
LightGBM	Light Gradient Boosting Machine
LoA	Limits of Agreement
LOSOCV	Leave-One-Subject-Out Cross Validation
MAE	Mean Absolute Error
MER	Mean Error Rate
OF	Optical Flow
PC	Principal Component
PCA	Principal Component Analysis
PCC	Pearson Correlation Coefficient
PPG	Photoplethysmography
RMSE	Root Mean Square Error
ROI	Region of Interest
rPPG	remote Photoplethysmography
SD	Standard Deviation
SNR	Signal-to-Noise Ratio
SOBI	Second-Order Blind Identification
SOTA	State of the Art
